# The Role of High-Density Lipoproteins in Endothelial Cell Metabolism and Diabetes-Impaired Angiogenesis

**DOI:** 10.3390/ijms21103633

**Published:** 2020-05-21

**Authors:** Khalia R. Primer, Peter J. Psaltis, Joanne T.M. Tan, Christina A. Bursill

**Affiliations:** 1 Faculty of Health and Medical Sciences, University of Adelaide, Adelaide, South Australia 5000, Australia; khalia.primer@sahmri.com (K.R.P.); peter.psaltis@sahmri.com (P.J.P.); joanne.tan@sahmri.com (J.T.M.T.); 2 Vascular Research Centre, South Australian Health and Medical Research Centre, Adelaide, South Australia 5000, Australia; 3 Centre for Nanoscale Biophotonics, Adelaide, South Australia 5000, Australia

**Keywords:** diabetes mellitus, angiogenesis, high-density lipoprotein, endothelial cell, metabolism, metabolic reprogramming

## Abstract

Diabetes mellitus affects millions of people worldwide and is associated with devastating vascular complications. A number of these complications, such as impaired wound healing and poor coronary collateral circulation, are characterised by impaired ischaemia-driven angiogenesis. There is increasing evidence that high-density lipoproteins (HDL) can rescue diabetes-impaired angiogenesis through a number of mechanisms, including the modulation of endothelial cell metabolic reprogramming. Endothelial cell metabolic reprogramming in response to tissue ischaemia is a driver of angiogenesis and is dysregulated by diabetes. Specifically, diabetes impairs pathways that allow endothelial cells to upregulate glycolysis in response to hypoxia adequately and impairs suppression of mitochondrial respiration. HDL rescues the impairment of the central hypoxia signalling pathway, which regulates these metabolic changes, and this may underpin several of its known pro-angiogenic effects. This review discusses the current understanding of endothelial cell metabolism and how diabetes leads to its dysregulation whilst examining the various positive effects of HDL on endothelial cell function.

## 1. Introduction

Diabetes mellitus is one of the most debilitating and prevalent diseases in the world and imposes a significant health and economic burden upon the global community. As of 2017, diabetes affects 425 million people worldwide. This number is expected to reach 629 million by 2045 [1]. The social and financial burden of the disease is staggering. Worldwide healthcare expenditure tops 850 billion USD, and this is predicted to leap to 958 billion USD by 2045. The vascular complications associated with diabetes are extremely diverse. Diabetes is an established risk factor for cardiovascular disease and contributes to multiple vascular complications, including neuropathy, retinopathy, nephropathy, impaired wound healing, and poor outcomes related to myocardial infarction (MI). All of these are associated with dysregulated angiogenesis. Successful wound repair and coronary collateral vessel formation post-MI are specifically dependent on appropriate pro-angiogenic responses to ischaemia, and there is emerging evidence that this is regulated by endothelial cell metabolism. Despite advances in therapeutic strategies for these vascular complications, many patients do not respond effectively to current treatments. This represents an unmet clinical need for the development of new therapies that rescue impaired ischaemia-driven angiogenesis in diabetes. Accumulating evidence supports high-density lipoproteins (HDL) as an exciting new therapeutic option. HDL can promote in vitro angiogenesis functions in high glucose conditions and augment ischaemia-driven angiogenesis in diabetic pre-clinical models [2,3]. This review will discuss in detail the multiple mechanisms for the pro-angiogenic effects of HDL in diabetes, including the emerging evidence for a central role in the correction of endothelial cell metabolism.

## 2. High-Density Lipoproteins

**Figure 1:**
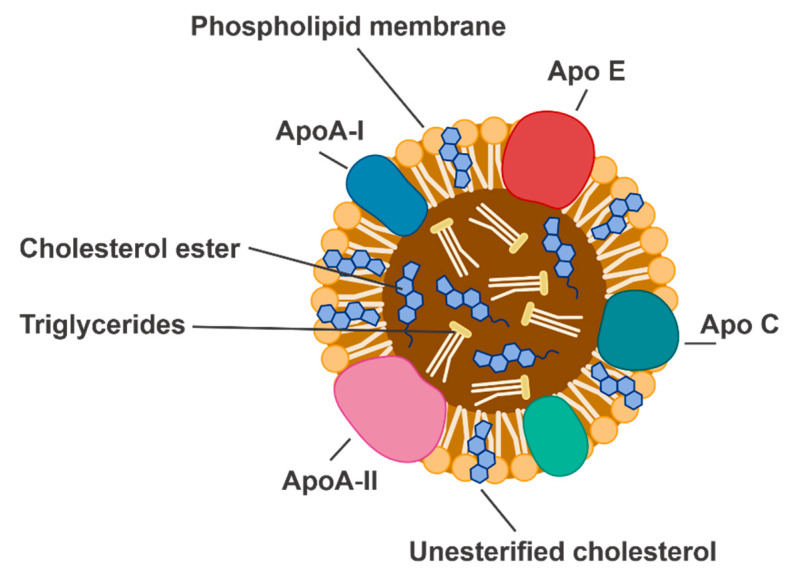
The structure of a high-density lipoprotein particle. Apo, apolipoprotein.

High-density lipoproteins (HDL) are highly heterogenous particles composed of an outer layer of apolipoproteins (apo) and phospholipids, surrounding a core of esterified cholesterol (Figure 1). ApoA-I is the predominant protein moiety in HDL and is thought to impart the many biological properties of HDL. HDL cholesterol (HDL-C) refers to the HDL particles in the bloodstream that are specifically carrying cholesterol. This is distinct from ‘HDL’, which refers to the particle itself, and which can have specific effects independent of cholesterol efflux and can be modified by various disease milieu [4]. It is necessary to distinguish between these two forms of notation, as this highlights the breadth of effects had by HDL. Additionally, investigating the role of HDL as a discrete particle independent of cholesterol efflux may identify reasons why HDL-C-raising therapies have demonstrated limited clinical benefit [4].

HDL primarily mediates reverse cholesterol transport by carrying cholesterol from peripheral tissues, including from macrophages in atherosclerotic plaques, to the liver for metabolism and excretion. This is a critical function that is a key contributor to the cardioprotective properties of HDL. HDL effluxes cholesterol by interacting with two cholesterol transporters ABCA1 and ABCG1 and the scavenger receptor SR-BI. ABCA1 interacts specifically with lipid-free apoA-I. Once apoA-I has acquired lipid to become a discoidal particle, it can then interact with ABCG1 and SR-BI. ABCA1 and ABCG1 contribute to the majority of the cholesterol efflux exchange but are also linked to a number of downstream signalling pathways, some of which are associated with angiogenesis. ABCG1, for example, mediates downstream signalling events that lead to elevated endothelial nitric oxide synthase (eNOS) activity, an important promoter of neovascularisation. This pathway is dependent on cholesterol efflux as it reduces the intracellular concentration of oxysterol 7-ketocholesterol [5]. Of the three cell-surface proteins that interact with HDL, SR-BI mediates the most downstream signalling pathways. These are either dependent or independent of cholesterol efflux. SR-BI is distinct to the other cholesterol transporters in that cholesterol can be exchanged bidirectionally between the cell and the HDL particle. Many of the signalling pathways downstream of SR-BI overlap significantly with those that regulate angiogenesis, including the phosphoinositide 3-kinase (PI3K)/Akt and MAPK pathways that will be discussed in more detail later in this review [6]. For the most part, apoA-I is believed to be the key component that mediates the multiple effects of HDL. The only other reported component of HDL that stimulates significant vascular biological effects is a sphingolipid, which interacts with the S1P1 receptor and activates downstream signalling events that are very similar to those downstream of SR-BI in endothelial cells and regulates angiogenesis [6]. It should be noted, however, that sphingolipid makes up only a tiny proportion of the HDL particle, and it has been suggested that its physiological relevance within HDL is likely to be limited.

HDL has been implicated in numerous diseases. These include rheumatoid arthritis (RA), a chronic inflammatory disorder of the joints that causes gradual destruction of the bones, which becomes increasingly painful for a patient. It has been reported that patients with RA have overall decreased HDL-C levels, but increased amounts of dysfunctional and pro-inflammatory HDL [7]. Similarly, metabolic syndrome is associated with decreased HDL-C levels, which is accompanied by obesity, hypertriglyceridaemia, insulin resistance, and impaired glucose tolerance [7,8]. HDL has also long been studied extensively in the context of vascular disease. Strong inverse relationships have been established between low levels of circulating HDL and the incidence of cardiovascular disease (CVD) [7,8,9]. However, the active, positive effect that HDL has on the cardiovascular system is complex and remains to be fully characterized.

## 3. Diabetes-Impaired Angiogenesis

Impaired ischaemia-driven angiogenesis is a hallmark of certain diabetic vascular complications, such as impaired wound healing and poor recovery following myocardial infarction. Angiogenesis is the protrusion of new vessels from existing ones into a tissue to form a mature vascular network that can re-oxygenate the ischaemic site. Across all diabetic vascular complications associated with impaired ischaemia-driven angiogenesis, epidemiological data show that increased HDL-C levels are associated with improved outcomes, or lower severity or incidence of the complication [7,10,11,12].

### 3.1. Wound Healing

Diabetic patients are particularly susceptible to developing persistent wounds that heal slowly and expose the patient to infection and further harm. Combined with other diabetic complications, such as peripheral neuropathy, which reduces nerve function and sensation in the extremities, impaired wound healing can lead to the development of diabetic foot ulcers (DFU). The pathophysiology of DFU is complex and multifactorial, and no therapy currently exists, which adequately addresses these complexities to achieve a clinical benefit. The current treatment strategy for DFU primarily involves the management of the patient’s co-morbidities that may contribute to ulcer development, such as glycaemic status or physical fitness. Additionally, regular dressing changes, management of infection, offloading and debridement of the wound tissue are employed to enhance the likelihood of a DFU healing correctly [13,14]. Outside of these standard-of-care approaches, there are very few effective therapies that actively improve the wound healing process.

Angiogenesis is a central component of wound healing. One reason for this is that neovessels deliver oxygen and nutrients to promote new tissue formation during the proliferative phase of healing [15]. Diabetic wounds exhibit decreased capillary density and vascularity due to insufficient angiogenesis [15,16]. Specifically, it has been shown that in chronic diabetic wounds, the angiogenic response normally induced by hypoxia is dysregulated [15,17]. Consistent with this, agents that inhibit angiogenesis, such as TNP-470 and SU5416, have been shown to inhibit wound repair [18,19]. Therefore, promoting wound angiogenesis presents a strategy to accelerate wound closure. This has been confirmed in murine wound healing studies using pro-angiogenic agents, such as vascular endothelial growth factor A (VEGFA) [20].

Whilst limited, there is epidemiological evidence that circulating HDL levels are inversely associated with the risk of DFU and amputation. For example, it has been reported that lower HDL levels are associated with increased DFU severity as classified by Wagner’s severity level [10], and are also subsequently associated with an increased incidence of lower extremity amputation and wound-related death [11]. This suggests that higher HDL levels may be protective against the pathophysiological development of chronic diabetic wounds.

### 3.2. Recovery Following Myocardial Infarction

In the context of myocardial infarction, ischaemia-driven angiogenesis is a critical component of coronary collateral circulation development. Chronic imbalances in myocardial oxygen supply and demand can lead to tissue ischaemia, which induces adaptive neovascularisation to increase oxygen supply to the myocardium. The extent to which a patient possesses coronary collateral circulation plays a critical role in determining how vulnerable they may be to athero-occlusive disease in the coronary arteries. A well-developed coronary collateral circulation is associated with improved cardiac function and survival following myocardial infarction (MI), as well as improved prognosis in the context of stable chronic coronary disease. Similar to wound healing, patients with diabetes have poorer coronary collateral circulation, which is associated with much worse outcomes following MI and revascularisation procedures [21,22,23].

The relationship between HDL and myocardial infarction has also been investigated. A study in the early 1980s examined the prognostic significance of HDL-C levels after patients had recovered from an acute MI. This study, conducted with data from the Coronary Drug Project, determined that low levels of HDL-C were associated with increased mortality following an acute MI [24]. Since then, additional studies have found supporting evidence that low levels of HDL-C or a high low-density lipoprotein cholesterol (LDL-C)/HDL-C ratio are common in patients with acute MI [25] and that these patients have a significantly higher in-hospital mortality rate [25,26] and cardiac mortality rate [26,27].

There is also an association between HDL and coronary collateral circulation development. In a retrospective cross-sectional study, Hasan et al. determined that low levels of HDL cholesterol were an independent predictor of poor coronary collateral circulation [12]. These results suggest a role for HDL levels in the pathophysiology of MI.

### 3.3. Dysregulated Angiogenesis in Diabetes

Within the complex pathology of diabetes mellitus, there are also a number of complications in which angiogenesis is upregulated or otherwise dysregulated. These include diabetic retinopathy (DR), which can lead to blindness, and diabetic nephropathy (DN), which can cause significant damage to the kidneys. The role of angiogenesis in these complications is very different from the role of impaired ischaemia-driven angiogenesis in wound healing and MI.

DR is characterised by two clinical stages, non-proliferative DR and proliferative DR (PDR). PDR represents a more advanced disease stage and is characterised by abnormal, increased neovascularisation of the retina. During this stage, the new abnormal vessels may leak into the vitreous fluid of the eye and cause serious impairment to the patient’s vision. Mechanistically, this increase in dysfunctional vessel formation is initially caused by retinal ischaemia which induces the VEGF pathway. This pathology is also heavily reliant on inflammatory pathways. Furthermore, current therapies for PDR are primarily anti-angiogenic, whilst therapies for impaired wound healing and myocardial ischaemia are pro-angiogenic [28].

DN presents another completely distinct pathology associated with dysregulated angiogenesis. DN is one of the leading causes of end-stage kidney disease in the world, and its development is complex. Excess hyperglycaemia-induced reactive oxygen species (ROS) cause damage to the kidney’s glomeruli, leading to excretion of albumin in the urine. This damage is not underpinned by excess or inadequate angiogenesis, but rather a dysfunction of the endothelial cells, podocytes, and mesangial cells which mediate glomerular filtration barrier function [29].

The differences between these complex pathologies highlight the breadth of effects diabetes has on the vasculature and demonstrates why the development of clinically-effective therapies is difficult. This review will focus on impaired ischaemia-driven angiogenesis, and specifically on the metabolism of endothelial cells found in cardiac vessels or associated with wound healing in the peripheral vasculature. We will discuss the disturbances caused by diabetes and the current evidence for how HDL regulates or corrects these pathways.

## 4. Physiological Angiogenesis and Its Regulation by HDL

Physiological ischaemia-driven angiogenesis is complex and regulated by many different factors. Endothelial cells are the main vessel-forming cells in angiogenesis and exist in three sub-types; migratory tip cells, proliferative stalk cells, and quiescent phalanx cells [30]. These sub-types are influenced by signalling molecules in a dynamic fashion to regulate cellular metabolism and behaviour, such as migration and proliferation.

Under normal conditions, endothelial cells are quiescent. This can be interrupted by a number of physiological factors to induce the shift to the migratory and proliferative phenotype critical for angiogenesis. One of these factors is hypoxia signalling. Hypoxia signalling is characterised by a decrease in the activity of the prolyl hydroxylase domain (PHD) proteins, which ordinarily utilise oxygen to target the hypoxia-inducible factors (HIF) for degradation [31]. To achieve this, the PHD proteins hydroxylate HIF subunits, which are then recognised by the von Hippel–Lindau (VHL) protein of the E3 ubiquitin ligase complex. The HIF subunits are then quickly degraded through the proteasomal degradation pathway [31]. Under conditions of low oxygen, the decrease in PHD protein activity allows for the HIF subunit HIF-1α to accumulate, rather than be degraded. HIF-1α then travels from the cytosol of a cell to the nucleus and, in combination with other subunits, initiates transcription of key angiogenic genes [32,33]. Potentially the most important of these is VEGFA, which is secreted by cells to create a signalling molecule concentration gradient. This gradient serves to induce endothelial cell proliferation and migration and leads them to the origin of hypoxia, forming new blood vessels as they travel [3,34]. In addition to this, fibroblast growth factors (FGFs) are also increased in response to hypoxia and contribute to increased endothelial cell proliferation in a similar way [35,36].

Other key contributors to this process are endothelial progenitor cells (EPCs). EPCs circulate in the bloodstream and respond to pro-angiogenic signals to differentiate into mature endothelial cells and contribute to neovascularization [37]. Combined, HIF-1α and VEGFA reprogram critical aspects of endothelial cell function to ensure their survival in hypoxia and the creation of new blood vessels.

Some HIF-1α-independent pathways and transcription factors have also been found to be critically important in angiogenesis. One of these is the forkhead box O (FOXO) transcription factor, FOXO1. FOXO1 is an effector of the PI3K/Akt pathway, and its nuclear activity can be inhibited by Akt-mediated phosphorylation. Wilhelm et al. demonstrated that FOXO1 acts as an enforcer of endothelial cell quiescence and that its deletion in mice causes an uncontrolled increase in vessel sprouting. Contrastingly, its overexpression severely restricted angiogenesis and led to vessel thinning [38].

Another important transcription coactivator is peroxisome proliferator-activated receptor gamma coactivator 1-alpha (PGC-1α). PGC-1α is well recognised as a central modulator of cellular metabolism and mitochondrial biogenesis. In muscle cells, PGC-1α has also been shown to potently upregulate VEGF under hypoxic conditions in a mechanism that is independent of HIF-1α. Arany et al. demonstrated this by exposing cultured muscle cells to a low oxygen environment. This induced the expression of PGC-1α, which then coactivated oestrogen-related receptor-α (ERR-α) on the *VEGF* promoter, leading to increased expression of VEGF and promotion of capillary density in skeletal muscle. This identified a role for PGC-1α in the regulation of angiogenic signalling, potentially in connection with its role in metabolism [39]. FOXO1 and PGC-1α are predominantly recognised as modulators of metabolism but are also clearly associated with angiogenesis.

HDL has been shown to regulate ischaemia-induced angiogenesis in a number of ways. An early study showed that in a murine model of hindlimb ischaemia, infusions of reconstituted HDL (rHDL, apoA-I complexed with phospholipid) increased the number of EPCs which homed to the ischaemic limb, resulting in improved reperfusion [40]. Mechanistically, in vitro studies demonstrated that rHDL promoted the differentiation of EPCs through the PI3K/Akt pathway [40]. Furthermore, studies conducted in patients with type 2 diabetes mellitus found that infusions of rHDL improved vascular function [41], and increased the number of circulating EPCs [42].

**Figure 2:**
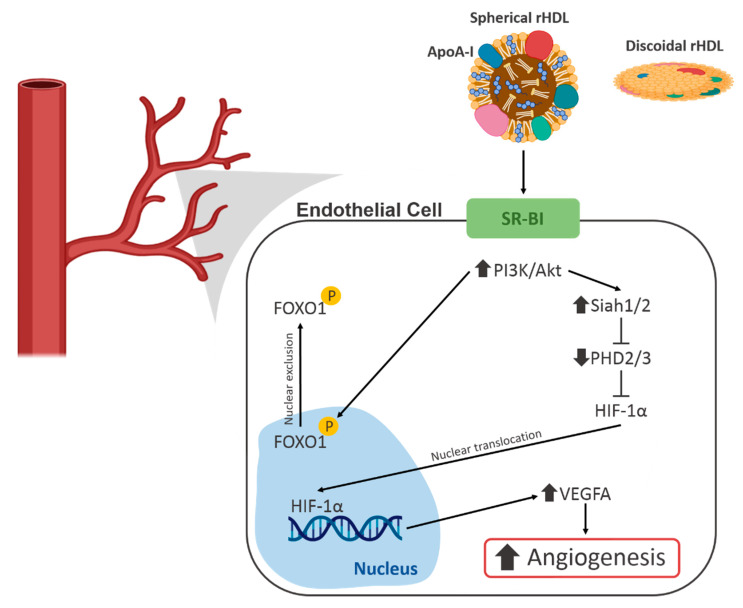
A schematic of the known effects of high-density lipoproteins (HDL) on angiogenic signalling pathways in endothelial cells. FOXO1, Forkhead Box O1; HIF-1α, hypoxia-inducible factor 1α; PHD, prolyl hydroxylase domain; PI3K, phosphoinositide 3-kinase; SR-BI, scavenger receptor B I.

Studies have also examined the effects of rHDL on the HIF-1α/VEGFA hypoxia signalling pathway, which initiates angiogenesis. Neovascularisation and blood flow reperfusion were increased by apoA-I infusions in the murine hindlimb ischaemia model. This study also examined the effect of rHDL on endothelial cell function in vitro and found that rHDL enhanced hypoxia-stimulated migration, proliferation, and tubulogenesis. It was then determined that these effects were mediated by augmentation of HIF-1α, VEGFA and VEGF receptor 2 (VEGFR2) [43]. It was demonstrated by a second study that these effects were due to changes in the post-translational modulation of HIF-1α induced by rHDL. In this study, Tan et al. found that through an interaction with scavenger receptor B I (SR-BI) and the PI3K/Akt pathway, rHDL increased the expression of the E3 ubiquitin ligases Siah1 and Siah2, which are responsible for targeting the PHD proteins for degradation. This resulted in decreased PHD protein activity, allowing HIF-1α to accumulate and promote transcription of VEGFA [2] (Figure 2).

There is also evidence that HDL regulates FOXO1 to positively effect endothelial cell function. A study by Theofilatos et al. demonstrated that treatment of human aortic endothelial cells with rHDL led to increased phosphorylation of FOXO1 by Akt, followed by its exclusion from the nucleus [44] (Figure 2). This resulted in increased expression of angiopoietin-like 4 (ANGPTL4), which is a potent pro-angiogenic molecule [45].

The exact mechanism by which HDL achieves these various salutary effects is complex and may be due to multiple factors. The effects on the HIF-1α/VEGFA signalling pathways have been shown to be dependent on an interaction between the rHDL particle and scavenger receptor class B type 1 (SR-BI) [3]. SR-BI plays a role in reverse cholesterol transport, and its presence is essential for rHDL to elicit its pro-angiogenic effects [3]. However, the role of cholesterol efflux was not directly addressed in these studies. Additionally, several studies referred to in this review have demonstrated positive effects on the vasculature or EPCs with only the apoA-I protein moiety of HDL. This indicates that there may be multiple mechanisms by which HDL and its apoA-I component are able to elicit positive effects on endothelial cell function.

Taken together, these studies highlight that rHDL regulates multiple aspects of pro-angiogenic signalling pathways to achieve a positive effect or ‘rescue’ of diabetes-impaired angiogenesis. However, given the complexity of diabetes and the associated vascular complications, it is necessary to fully understand the breadth of effects had by rHDL to develop it as a potential therapeutic agent. The HIF-1α/VEGFA hypoxia signalling pathway is well-characterised and considered the main driver of ischaemia-induced angiogenesis. HDL has been implicated in multiple steps of this pathway. The response elicited by this pathway in endothelial cells is complex, and an orchestration of many different factors is required for the cells to respond to hypoxia and participate in angiogenesis correctly. Endothelial cell metabolism and the pathways which reprogram it in response to hypoxia are fast emerging as drivers of angiogenesis in their own right. HDL is also emerging as a regulator of this, supporting its already diverse effects on endothelial cells and angiogenesis.

## 5. Endothelial Cell Metabolism

**Figure 3:**
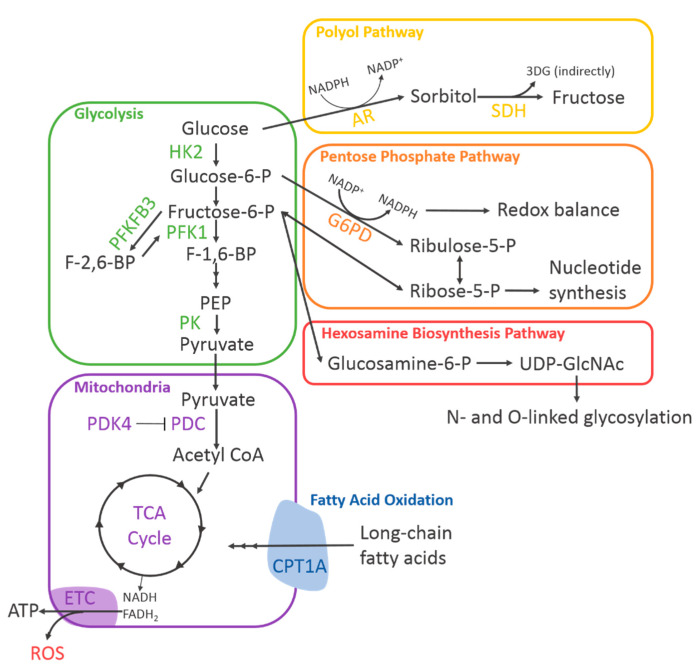
Diagram of endothelial cell metabolism. A simplified diagram of endothelial cell metabolism depicting the known metabolic pathways and their rate-limiting enzymes. 3DG, 3-deoxyglucosone; AR, aldose reductase; CPT1A, carnitine palmitoyltransferase 1A; ETC, electron transport chain; G6PD, glucose-6-phosphate dehydrogenase; HK2, hexokinase 2; NADPH, nicotinamide adenine dinucleotide phosphate; PDC, pyruvate dehydrogenase complex; PDK4, pyruvate dehydrogenase kinase 4; PFK1, phosphofructokinase 1; PFKFB3, phosphofructokinase-2/fructose-2,6-bisphosphatase isoform 3; PK, pyruvate kinase; SDH, sorbitol dehydrogenase; UDP-GlcNAc, uridine diphosphate n-acetylglucosamine.

To support their wide range of critical functions, endothelial cells possess unique metabolic capabilities. Without metabolism there can be no energy production or replenishment of nutrient pools, and without this, an endothelial cell cannot fulfil any of its necessary functions. To understand how we can target endothelial cell metabolism to have a positive impact on diabetes-impaired angiogenesis, we must first understand its regulatory mechanisms (Figure 3).

### 5.1. Glycolysis

Glycolysis describes a chain of reactions by which one molecule of glucose is broken down into two molecules of pyruvate. Glycolysis occurs in the cytosol, does not require oxygen, and can, therefore, occur in the presence or absence of oxygen. There are many steps in this chain of reactions, one of which is ATP-producing. Glycolysis produces approximately two molecules of ATP for every molecule of glucose. There are also many metabolic intermediates produced as glycolysis progresses, and these have their own roles in the regulation of endothelial cell function, which will be discussed later.

Active endothelial cells rely on glycolysis for the bulk of their ATP production, though this may seem counter-intuitive, given their ordinarily easy access to oxygen from the bloodstream [30]. However, the sheer quantity of oxygen that endothelial cells are exposed to could easily lead the cells to experience oxidative stress if they did not restrict flux through pathways that consume oxygen. Furthermore, reducing reliance on oxygen-consuming pathways ensures that endothelial cells are always primed to function in hypoxia, which is useful for revascularisation of ischaemic tissues. This is in contrast to many other cell types for which full oxidation of glucose through mitochondrial respiration is the most efficient method of ATP production.

Upon encountering hypoxia, endothelial cells undergo a substantial metabolic shift. It is essential that endothelial cells further upregulate glycolysis to account for the increased energy demands of proliferation and migration, whilst simultaneously keeping oxygen consumption low to avoid oxidative stress. To achieve this, VEGF and FGF signalling support an increase in glycolytic flux. This means that the rate of glucose breakdown through glycolysis increases, producing more ATP, more glycolytic intermediates, and more pyruvate. This section will discuss the key roles of VEGF and FGFs in regulating glycolytic flux.

First, the increase in glycolytic flux has been found to be mediated, at least in part, by an interaction between VEGF and 6-phosphofructo-2-kinase/fructose-2,6-biphosphatase 3 (PFKFB3) [30]. An important rate-limiting step in glycolysis is the conversion of fructose-6-phosphate (F6P) to fructose-1,6-bisphosphate (F1,6P_2_) by 6-phosphofructo-1-kinase (PFK-1). PFKFB3 synthesises fructose-2,6-bisphosphate (F2,6P_2_), a strong activator of PFK-1. Therefore, the activity of PFKFB3 represents an avenue for potent upregulation of glycolysis. De Bock et al. have shown that PFKFB3 knockdown using short hairpin RNA (shRNA) reduced glycolytic flux by 35% in both microvascular and arterial endothelial cells, indicating that PFKFB3 is important for maintaining adequate levels of glycolytic flux in endothelial cells. Importantly, it was shown that VEGF increases both PFKFB3 expression and glycolysis. With respect to the effect on angiogenesis, knockdown of PFKFB3 decreased vessel sprouting in endothelial cell spheroids. Further investigation of this in vivo revealed that mice with endothelial cells deficient in PFKFB3 displayed significant defects in retinal blood vessel development as well as decreased vascular area in the hindbrain [30].

Other glycolytic enzymes have also been implicated in the regulation of endothelial cell angiogenic functions. One of these is hexokinase 2 (HK2), which phosphorylates a molecule of glucose to produce glucose-6-phosphate. This is the first step in glycolysis and is also rate-limiting. Yu et al. investigated the relationship between FGF2 and HK2 in endothelial cells with the aim of delineating the mechanism by which FGF2 stimulates angiogenesis. This group found that mouse embryos deficient in endothelial FGF receptor 1 (FGFR1) exhibited reduced vessel branching in the skin. To elucidate this mechanism, human umbilical vein endothelial cells (HUVECs) were treated with FGF, which significantly enhanced glycolysis and HK2 expression. Knockdown of HK2 significantly reduced glycolysis, whilst adenoviral-mediated overexpression increased glycolysis. This study identified a relationship between FGF and HK2 in endothelial cells and determined that HK2-mediated glycolysis was essential for angiogenesis [36].

Pyruvate kinase (PK) catalyses the conversion of phosphoenolpyruvate to pyruvate, which is the final rate-limiting step in glycolysis. When PK activity is low, upstream glycolytic intermediates may accumulate and be shunted to various side-pathways. Endothelial cells predominantly express the PKM2 isoform of this enzyme, and this was examined in the context of angiogenesis by Kim et al. This group knocked down PKM2 using siRNA and observed significant suppression of cell proliferation, migration, and tubule formation in vitro. This was associated with a decrease in extracellular acidification, which represents the completion of the glycolytic pathway. Furthermore, endothelial cell-specific deletion of PKM2 in mice was associated with a significant reduction in retinal vessel density and branching. Finally, PKM2 was found to achieve its effects on the proliferation via inhibition of p53, causing a blockade of the cell cycle. Although this role of PKM2 was found to be independent of its enzymatic activity, this study nevertheless highlights the close relationship between the integrity of metabolic pathways and the angiogenic capacity of endothelial cells [46].

Together, this research highlights the importance of glycolysis in angiogenesis and identifies key regulatory mechanisms that may be implicated in diabetes-impaired angiogenesis (Figure 3).

### 5.2. Mitochondrial Respiration and Hypoxia Tolerance

It has also been shown that endothelial cells maintain very low levels of mitochondrial respiration relative to their rate of glycolysis [30]. Mitochondrial respiration, which encompasses both the tricarboxylic acid (TCA) cycle and oxidative phosphorylation, ordinarily consumes a high quantity of oxygen and subsequently contributes to the production of reactive oxygen species (ROS). Suppressing oxidative phosphorylation may be necessary for endothelial cells to reduce ROS production and maintain redox homeostasis.

Recently, a key regulatory step has been identified, which may be critical for adequate suppression of mitochondrial respiration in endothelial cells. The pyruvate–dehydrogenase complex (PDC) catalyses the conversion of pyruvate to acetyl coenzyme A (CoA) so that it may condense with oxaloacetate to enter the TCA cycle and contribute to mitochondrial respiration [47]. The pyruvate dehydrogenase lipoamide kinases (PDK) of which there are four subtypes, PDK1-4, are particularly important as they inhibit the PDC. The PDKs inhibit the PDC by phosphorylating three serine residues in the E1α subunit of the PDC in a tissue-specific manner [47,48]. This decreases the amount of acetyl CoA produced from glucose-derived pyruvate, which fuels the TCA cycle, thereby reducing the rate of respiration.

The importance of PDK4 in hypoxia-induced metabolic reprogramming was established by Aragones et al. when they demonstrated that myofibers deficient in PHD1, which normally degrades HIF proteins, exhibited elevated levels of PDK4. These myofibers were significantly more tolerant of hypoxia, consumed less oxygen, and were experiencing less oxidative stress [49]. Whilst Aragones et al. did observe that this protection against oxidative stress was independent of increased angiogenesis, it does demonstrate a direct mechanistic link between hypoxia signalling and PDK4. Consistent with this, our group has demonstrated that PDK4 was increased in human coronary artery endothelial cells (HCAECs) exposed to hypoxia. Furthermore, PDK4 knockdown by short interfering RNA (siRNA) significantly impaired endothelial cell tubule formation and migration in vitro [50]. This indicated that suppression of glucose-driven mitochondrial respiration might be essential for maintaining the endothelial cell capacity for angiogenesis, potentially by decreasing mitochondrial production of ROS or allowing for compensatory increases in fatty acid oxidation (Figure 3).

### 5.3. Fatty Acid Oxidation

Fatty acid oxidation (FAO) represents an alternate avenue for the production of acetyl CoA from long-chain fatty acids and bypasses the PDC. This is achieved by direct production of acetyl CoA from long-chain fatty acids, which can then condense with oxaloacetate to form citrate and fuel the TCA cycle. In endothelial cells, specifically, the main mechanism which regulates FAO involves AMP-activated protein kinase (AMPK), which is activated by hypoxia [51]. AMPK indirectly activates carnitine palmitoyl transferase 1A (CPT1A), which shuttles long-chain fatty acids into the mitochondria and represents a rate-limiting step of FAO [52].

Whilst FAO can be used for the generation of ATP through mitochondrial respiration, endothelial cells rely predominantly on glycolysis for energy production. Recent research does, however, point to FAO as an essential contributor to endothelial cell functions beyond energy production.

Kalucka et al. examined the metabolism of quiescent endothelial cells. This group found that quiescent endothelial cells upregulated FAO three times more than proliferative endothelial cells and that this was primarily in support of nicotinamide adenine dinucleotide phosphate (NADPH) regeneration for redox homeostasis. Blocking FAO in these cells induced significant oxidative stress, whilst supplementing them with acetate rescued this. This demonstrates that FAO is essential for maintaining redox balance in quiescent endothelial cells [53].

In contrast, Schoors et al. investigated FAO in proliferative endothelial cells undergoing vessel sprouting, again with a focus on identifying an alternative role for the process beyond energy production. Schoors et al. knocked down CPT1A and found that this impaired vessel sprouting in endothelial cell spheroids due to decreased cell proliferation. CPT1A knockdown did not lower ATP levels, and only increased ROS levels by approximately 20%, a level thought to have a positive effect on endothelial cell proliferation. It was then determined that acetyl CoA from FAO was cycling through the TCA cycle and contributing to de novo synthesis of deoxyribonucleotides in endothelial cells, and that knockdown of CPT1A reduced this and subsequently impaired de novo DNA synthesis. This culminated in the impairment of endothelial cell proliferation and overall vessel sprouting [54]. This contrasts with the role of FAO in maintaining redox balance in quiescent endothelial cells but nevertheless suggests that FAO is important for correct endothelial cell function and angiogenesis.

### 5.4. The Pentose Phosphate Pathway

Glycolytic side pathways are also critical for endothelial cell function. These involve various glycolytic intermediates and have a wide range of functions that can be either positive or negative for an active endothelial cell. Thus far, the pentose phosphate pathway (PPP) has been demonstrated to be the most important of these side pathways. The PPP utilises glucose-6-phosphate from glycolysis in two different branching pathways. The oxidative branch (oxPPP) comprises an irreversible reaction which generates NADPH and ribose-5-phosphate (R5P), whilst the non-oxidative branch produces only R5P [55]. NADPH is critical for redox homeostasis as it allows for the conversion of oxidised glutathione (GSSG) to its reduced form (GSH). Reduced glutathione is an essential antioxidant which neutralises reactive oxygen species [56]. Therefore, this mechanism is likely to play an important role in maintaining the redox balance of an endothelial cell during hypoxia-driven angiogenesis. Additionally, the R5P produced by this pathway is essential for the synthesis of nucleotides, which likely supports the increased proliferation required during vessel sprouting. The rate of the oxPPP is determined by glucose-6-phosphate dehydrogenase (G6PD). The importance of this pathway and its close relationship with the rate of glycolysis implicates it in diabetes-impaired angiogenesis.

## 6. Endothelial Cell Metabolism and Diabetes-Impaired Angiogenesis

The prevailing hypothesis regarding diabetes-impaired angiogenesis is that hyperglycaemia causes increased production of reactive oxygen species (ROS), which leads to general endothelial cell dysfunction. However, this is only one aspect of the complex pathology. Diabetic hyperglycaemia also negatively affects individual transcription factors, signalling molecules, and metabolic enzymes, which contribute to endothelial cell dysfunction and impaired angiogenesis. The HIF-1α/VEGFA hypoxia signalling pathway is a clear example of this. Furthermore, ROS production is closely tied to cellular metabolism, as we have discussed previously. Therefore, changes in cellular metabolism elicited by diabetes potentially underpin the increase in ROS production. Mitochondrial respiration is a major source of ROS, and several glycolytic side pathways contribute to cellular redox balance. The relationships between these pathways are complex, and it is difficult to tell from where the negative effects on angiogenesis stem.

### 6.1. Diabetes Impairs Central Metabolic Pathways

Angiogenesis is initiated by the HIF-1α/VEGFA hypoxia signalling pathway, which has a myriad of complex downstream effects that orchestrate the vessel sprouting and maturation process. It has been consistently demonstrated that hyperglycaemia or in vitro high glucose conditions negatively affect the expression of HIF-1α and its translocation to the nucleus [3]. This effect was found to be due to a high glucose-induced increase in the PHD proteins, which tag HIF-1α for degradation. Increased degradation of HIF-1α means it cannot travel to the nucleus and induce the transcription of pro-angiogenic genes. This, therefore, also impairs the hypoxia-induced expression of VEGFA and is subsequently associated with the impairment of angiogenesis under high glucose conditions [3].

**Figure 4:**
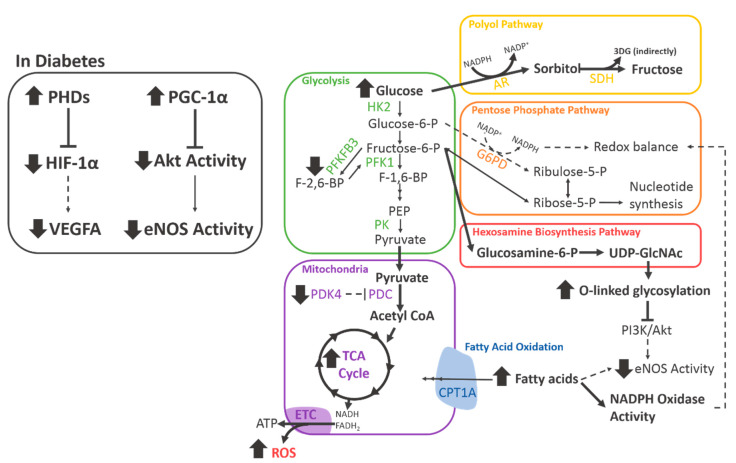
The effects of diabetes on endothelial cell metabolism. A diagram depicting the changes in endothelial cell metabolism elicited by diabetes. Bold lines indicate an increase, dashed lines indicate a decrease. 3DG, 3-deoxyglucosone; AR, aldose reductase; CPT1A, carnitine palmitoyltransferase 1A; ETC, electron transport chain; eNOS, endothelial nitric oxide synthase; G6PD, glucose-6-phosphate dehydrogenase; HIF-1α, hypoxia-inducible factor 1α; HK2, hexokinase 2; NADPH, nicotinamide adenine dinucleotide phosphate; PDC, pyruvate dehydrogenase complex; PDK4, pyruvate dehydrogenase kinase 4; PFK1, phosphofructokinase 1; PFKFB3, phosphofructokinase-2/fructose-2,6-bisphosphatase isoform 3; PHDs, prolyl hydroxylase domain proteins; PI3K, phosphoinositide 3-kinase; PK, pyruvate kinase; SDH, sorbitol dehydrogenase; UDP-GlcNAc, uridine diphosphate N-acetylglucosamine; VEGF, vascular endothelial growth factor. Bold arrows indicate an increase, dashed arrows indicate a decrease.

These negative effects on the central HIF-1α/VEGFA signalling pathway are also associated with downstream impairments to hypoxia-induced metabolic reprogramming. VEGFA is known to increase PFKFB3 expression in endothelial cells to support adequate upregulation of glycolysis in hypoxia. The effect of hyperglycaemia on PFKFB3 was tested by Rudnicki et al., who demonstrated that high glucose reduced the mRNA levels of *Pfkfb3* in endothelial cells isolated from mice [57] (Figure 4).

Suppression of mitochondrial respiration is also a critical aspect of metabolic reprogramming in response to hypoxia [49]. The expression of PDK4, which inactivates the PDC in endothelial cells, is known to be increased in response to hypoxia [58]. Recent studies by our group show that, importantly, this induction is impaired when the endothelial cells are exposed to high glucose, indicating a failure of the metabolic reprogramming response. These changes were also associated with a marked increase in mitochondrial respiration, which is a known source of ROS, as well as impaired in vitro angiogenesis [50].

PGC-1α is an important mediator of mitochondrial biogenesis and metabolism and is significantly affected by diabetes. Sawada et al. aimed to fully characterise the effect of diabetes on PGC-1α and determine how this affected endothelial cell function and angiogenesis. First, they demonstrated that PGC-1α was significantly elevated in endothelial cells from the heart or lung tissue from several different diabetic mouse models [59]. This effect was replicated in vitro with cultured primary endothelial cells exposed to high glucose. Furthermore, endothelial cells overexpressing PGC-1α exhibited significantly impaired migration, as demonstrated with a transwell assay. Mechanistically, PGC-1α was found to inhibit Akt phosphorylation of eNOS, which is essential for angiogenesis [59]. This study identified a strong link between the modulation of metabolism and control of angiogenesis, as well as highlighting the negative effects of diabetes on this mechanism.

Combined, these negative effects of diabetes affect central metabolic pathways and mediators, which normally support angiogenesis.

### 6.2. Glycolytic Side-Pathways

In addition to its negative effects on hypoxia signalling, hyperglycaemia also increases ROS production through a number of metabolic pathways.

The oxidative branch of the pentose phosphate pathway has been examined in this context, and it is well established that high glucose inhibits the rate-limiting enzyme of the oxPPP, glucose-6-phosphate dehydrogenase (G6PD) [60]. This blocks production of NADPH, which is essential for converting oxidised glutathione (GSSG) to its reduced form (GSH), thereby reducing the ability of the cells to adequately neutralise ROS [60]. This mechanism severely impairs the ability of endothelial cells to maintain redox balance in diabetes.

Once a cell begins to accumulate ROS through this mechanism, an additional cascade of events begins. Excess ROS damages a cell’s DNA, which can lead to aberrant transcription of various proteins. One specific protein that is aberrantly activated by ROS-induced DNA damage in endothelial cells is poly-ADP-ribose polymerase 1 (PARP1) [61]. PARP1 post-translationally modifies proteins to inactivate them, and one relevant target is the glycolytic enzyme glyceraldehyde-3-phosphate dehydrogenase (GAPDH) [61,62,63]. When GAPDH is inactivated in this manner, it causes a blockade in the glycolytic pathway which allows for the accumulation of intermediates that cannot flow further through glycolysis. With the known effect of hyperglycaemia on G6PD keeping these intermediates from being shunted to a useful pathway, such as the oxPPP, the intermediates instead increase flux through upstream pathways which are detrimental to cellular function and known to be increased in diabetic endothelial cells: the polyol pathway and hexosamine biosynthesis pathway (HBP).

In diabetes, excess glucose is shunted into the polyol pathway and converted to sorbitol by aldose reductase, a reaction which uses NADPH [64]. This increased usage of NADPH may further exhaust a cell’s ability to replenish the antioxidant GSH and exacerbate oxidative stress. Sorbitol is then converted to fructose by sorbitol dehydrogenase. This reaction also indirectly generates 3-deoxyglucosone (3DG) through the hydrolysis of the intermediate fructose-3-phosphate [65]. 3DG is a highly reactive compound known to contribute to the production of advanced glycated end-products (AGEs) [66,67]. AGEs are proteins or lipids that have been post-translationally glycated and are implicated in many aspects of diabetes-induced EC dysfunction. They have been shown to specifically inhibit angiogenesis by increasing extracellular matrix degradation by matrix metalloproteinases [68,69]. To conclude, increased flux through the polyol pathway caused by hyperglycaemia exacerbates oxidative stress and contributes to impaired angiogenesis.

One of the upstream glycolytic metabolites blocked by GAPDH inactivation is fructose-6-phosphate (F6P). F6P enters the hexosamine biosynthesis pathway and is converted to uridine diphosphate N-acetylglucosamine (UDP-GlcNAc), which is an essential substrate for N-linked and O-linked glycosylation of proteins. This post-translational modification of proteins is normally very important for normal cellular function. However, under hyperglycaemic conditions, excess F6P produced from glucose leads to aberrantly increased O-linked glycosylation of target proteins. Federici et al. examined this pathway in endothelial cells and found that O-linked glycosylation was significantly increased in HCAECs exposed to high glucose conditions [70]. This was found to impair PI3K/Akt signalling, which was then associated with a decrease in eNOS phosphorylation by Akt. Phosphorylation of eNOS increases the production of nitric oxide, which is pro-angiogenic [71,72]. A similar study by Luo et al. supported these findings by demonstrating that O-GlcNAc levels were increased in a murine streptozotocin model of diabetes and that this was indeed associated with impaired angiogenesis in an aortic ring assay. The group also showed that increased O-GlcNAc leads to reduced endothelial cell migration and tubule formation in vitro. Importantly, overexpression of O-GlcNAcase, a protein that reverses O-linked glycosylation, rescued migration and tubule formation of endothelial cells [72]. This indicates that increased flux through the HBP caused by hyperglycaemia impairs the PI3K/Akt signalling pathway, which has negative consequences for angiogenesis.

### 6.3. Diabetes and Fatty Acid Oxidation

The role of FAO in diabetes is complex and varies across tissue and cell types. It has been determined that FAO is essential for angiogenesis through its support of endothelial cell proliferation and biomass synthesis [54] Additionally, FAO is essential for the maintenance of quiescence and redox homeostasis in quiescent endothelial cells [53]. One additional aspect to consider is that whilst diabetes is characterised by hyperglycaemia, it is also associated with high circulating levels of free fatty acids (FFAs). Several studies have examined the effect of elevated FFAs on general endothelial function. Steinberg et al. investigated this in healthy patients with experimentally increased FFA levels and observed that the increase in FFAs was associated with endothelial dysfunction [73]. In a subsequent study, the same research group demonstrated that elevation of FFAs impaired endothelial insulin-mediated vasodilation and endothelial nitric oxide production [74]. This result was also demonstrated by Vigili et al., who showed that elevated FFAs impaired endothelium-dependent vasodilation [75]. One study has examined the effect of high glucose and elevated FFAs on ROS production in endothelial cells in vitro. Inoguchi et al. found that both high glucose and elevated FFAs increased superoxide production by NADPH oxidases in a protein kinase C-dependent manner [76]. This has some relevance for angiogenesis, given what is known about the necessity to regulate ROS production in a hypoxic environment and highlights the importance of the oxPPP in generating NADPH for maintaining redox balance. Whilst these effects do not necessarily directly reflect changes in angiogenesis, they do provide some insight into the general endothelial dysfunction caused by FFA elevation in patients with diabetes. It is necessary to investigate these effects further to understand how angiogenesis may be affected by elevated FFAs, and whether diabetes negatively affects FAO in endothelial cells.

These studies demonstrate that the effects of diabetes on endothelial cell metabolism are wide-ranging, complex, and significantly impair various angiogenic functions (Figure 4).

## 7. Emerging Role of HDL in Mechanisms of Endothelial Cell Metabolic Reprogramming and Diabetes-Impaired Angiogenesis

Earlier in this review, we highlighted the various positive effects of HDL on the cardiovascular system and angiogenesis in response to ischaemia and in diabetes. We have also established the importance of endothelial cell metabolic reprogramming as a driver of angiogenesis, and the various negative effects had upon its regulation by diabetes. Multiple lines of evidence point to a role for HDL in the regulation of endothelial cell metabolism in the context of diabetes-impaired angiogenesis.

### 7.1. HDL and Hypoxia Signalling

There is much evidence supporting the concept that HDL augments diabetes-impaired HIF-1α activity and VEGFA expression, resulting in the rescue of angiogenesis both in vitro and in vivo.

Specifically, Tan et al. found that rHDL rescued diabetes-impaired angiogenesis in two murine models of hindlimb ischaemia and wound healing. rHDL was shown to stabilise HIF-1α in high glucose by suppressing the PHD2 and three proteins, leading to an increase in the expression of VEGFA and rescuing high glucose-impaired tubulogenesis in vitro [3].

VEGF is one of the most important downstream targets of HIF-1α, and its expression is known to be impaired in diabetes [3]. The relationship between VEGF and glycolytic protein PFKFB3 has been well documented and is also essential for angiogenesis [30]. PFKFB3 expression is also impaired under high glucose conditions, which may contribute to negative effects on angiogenesis. rHDL is known to rescue diabetes-impaired expression of VEGFA [3], and this may also lead to the support of increased glycolytic flux in hypoxia and underpin some of the known pro-angiogenic activity of rHDL.

Furthermore, our research group has recently determined that PDK4, which inhibits mitochondrial respiration, is a target of rHDL. In human coronary artery endothelial cells, we have demonstrated that PDK4 expression is increased in hypoxia, but that this induction is impaired by exposure to high glucose conditions [50]. This aligns with the impairment to HIF-1α signalling seen under diabetic conditions in previous studies. Pre-incubation of the endothelial cells with rHDL restored the induction of PDK4 under high glucose and hypoxic conditions. When the oxygen consumption of the cells was examined as a measure of mitochondrial respiration, it was found that high glucose significantly increased oxygen consumption, but that pre-incubation with rHDL was able to return these levels to the baseline. These effects were also associated with the rescue of high glucose-impaired endothelial cell migration and tubulogenesis under hypoxic conditions [50]. We, therefore, propose that rHDL augments metabolic reprogramming via the upregulation of PDK4, decreasing oxygen consumption and potentially protecting the cells against the development of oxidative stress in hypoxia.

### 7.2. HDL and PI3K/Akt Signalling

Diabetes also affects endothelial cell metabolism via impairment of PI3K/Akt signalling. Hyperglycaemia causes the increased flux of fructose-6-phosphate through the hexosamine biosynthesis pathway, leading to dysregulation of O-linked glycosylation. One of the targets of this dysregulated glycosylation is the PI3K/Akt signalling pathway. This signalling pathway controls a number of critical mechanisms, including phosphorylation of eNOS by Akt. In diabetes, this pathway was significantly impaired in in vitro and in vivo models, and was associated with impaired angiogenesis [72]. A number of studies have demonstrated that rHDL can increase PI3K/Akt signalling; therefore, this is another pathway by which rHDL may support endothelial cell function and rescue angiogenesis in diabetes. One study demonstrated that intravenous injections of rHDL promoted differentiation of endothelial progenitor cells by increasing phosphorylation of Akt by PI3K in peripheral mononuclear cells. This was associated with augmentation of blood flow and increased capillary density in ischaemic limbs [40]. Another study demonstrated that the pre-incubation of endothelial cells with rHDL significantly increased protein expression of both PI3K and Akt under high glucose conditions [3]. This was subsequently associated with a significant increase in phosphorylation of eNOS by Akt and the rescue of high glucose-impaired tubulogenesis in vitro [3]. Furthermore, specific inhibition of PI3K/Akt with LY294002 in endothelial cells attenuated the augmentation of in vitro tubulogenesis, and HIF-1α and VEGFA by rHDL in response to hypoxia [2]. Finally, it has been demonstrated that rHDL initiates the nuclear exclusion of FOXO1 by increasing its phosphorylation by Akt, which has a number of positive effects on endothelial cell function [44].

**Figure 5:**
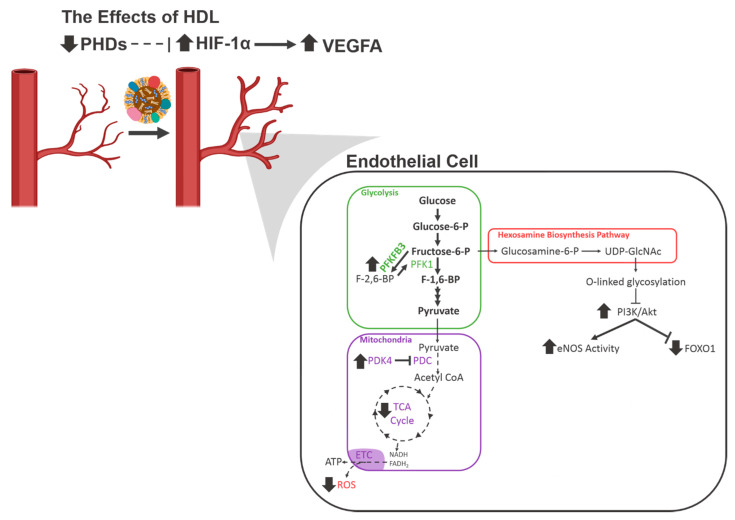
The known and proposed effects of HDL on endothelial cell metabolism. A diagram depicting the changes in endothelial cell metabolism caused by HDL under conditions of high glucose and hypoxia. Bold lines indicate an increase, dashed lines indicate a decrease. ETC, electron transport chain; eNOS, endothelial nitric oxide synthase; FOXO1, forkhead box O1; HIF-1α, hypoxia-inducible factor 1α; PDC, pyruvate dehydrogenase complex; PDK4, pyruvate dehydrogenase kinase 4; PHDs, prolyl hydroxylase domain proteins; PI3K, phosphoinositide 3-kinase; UDP-GlcNAc, uridine diphosphate N-acetylglucosamine; VEGF, vascular endothelial growth factor.

These results demonstrate that high-density lipoproteins affect multiple aspects of endothelial cell metabolism to achieve a pro-angiogenic effect under diabetic conditions (Figure 5). Several potential downstream targets of HDL have also been identified and represent avenues for further study. 

## 8. Conclusions

The dysregulated angiogenesis associated with diabetes mellitus presents a complex contribution to the pathology of diabetic vascular complications and the associated mortality. Therapies to correct this dysregulation are severely lacking, as is our understanding of the complexities of this process. Endothelial cell metabolism is a central driver of angiogenesis, but many aspects of metabolic reprogramming are negatively affected by diabetes, though our knowledge of this is still developing. HDL continues to represent a promising avenue for therapeutic development, as its multiple protective functions culminate in the rescue of diabetes-impaired angiogenesis and improved healing of chronic diabetic wounds. However, due to the relative lack of clinical success with HDL-raising therapies, further research must be conducted to fully elucidate the mechanisms involved in these processes and identify the best approach for harnessing the protective effects of HDL.

## Abbreviations

3DG	3-deoxyglucosone
AGE	Advanced glycation end products
AMPK	AMP-activated protein kinase
ANGPTL4	Angiopoietin-like 4
Apo	Apolipoprotein
CoA	Coenzyme A
CPT1A	Carnitine Palmitoyltransferase 1A
CVD	Cardiovascular disease
DFU	Diabetic foot ulcer
DN	Diabetic nephropathy
DR	Diabetic retinopathy
eNOS	Endothelial nitric oxide synthase
EPC	Endothelial progenitor cell
ERRα	Oestrogen-related receptor α
F1,6P2	Fructose-1,6-bisphosphate
F2,6P2	Fructose-2,6-bisphosphate
F6P	Fructose-6-phosphate
FAO	Fatty acid oxidation
FFA	Free fatty acid
FGF	Fibroblast growth factor
FGFR1	Fibroblast growth factor receptor 1
FOXO	Forkhead box O
G6PD	Glucose-6-phosphate dehydrogenase
GAPDH	Glyceraldehyde-3-phosphate dehydrogenase
GSH	Reduced glutathione
GSSG	Oxidised glutathione
HBP	Hexosamine biosynthesis pathway
HCAEC	Human coronary artery endothelial cell
HDL	High-density lipoprotein
HDL-C	High-density lipoprotein cholesterol
HIF	Hypoxia-inducible factor
HIF-1α	Hypoxia-inducible factor 1α
HK2	Hexokinase 2
HUVEC	Human umbilical vein endothelial cell
LDL	Low-density lipoprotein
LDL-C	Low-density lipoprotein cholesterol
MAPK	Mitogen-activated protein kinase
MI	Myocardial infarction
NADPH	Nicotinamide adenine dinucleotide phosphate
PARP1	Poly (ADP-Ribose) Polymerase 1
PDC	Pyruvate dehydrogenase complex
PDK	Pyruvate dehydrogenase kinase
PDK4	Pyruvate dehydrogenase kinase 4
PDR	Proliferative diabetic retinopathy
PFK-1	Phosphofructokinase-1
PFKFB3	6-phosphofructo-2-kinase/fructose-2,6-biphosphatase 3
PGC-1α	Peroxisome proliferator-activated receptor gamma co-activator 1α
PHD	Prolyl hydroxylase domain
PI3K	Phosphoinositide 3-kinases
PK	Pyruvate kinase
PPP	Pentose phosphate pathway
R5P	Ribose-5-phosphate
RA	Rheumatoid arthritis
rHDL	Reconstitute high-density lipoprotein
ROS	Reactive oxygen species
shRNA	Short hairpin RNA
siRNA	Small interfering RNA
SR-BI	Scavenger receptor BI
TCA	Tricarboxylic acid
UDP-GlcNAc	Uridine diphosphate N-acetylglucosamine
VEGFA	Vascular endothelial growth factor A
VEGFR2	Vascular endothelial growth factor receptor 2
VHL	Von Hippel-Lindau
